# Identification of differentially expressed miRNAs in individual breast cancer patient and application in personalized medicine

**DOI:** 10.1038/oncsis.2016.4

**Published:** 2016-02-15

**Authors:** F Peng, Y Zhang, R Wang, W Zhou, Z Zhao, H Liang, L Qi, W Zhao, H Wang, C Wang, Z Guo, Y Gu

**Affiliations:** 1 Department of Systems Biology, College of Bioinformatics Science and Technology, Harbin Medical University, Harbin, China; 2 Department of Pharmacology, Harbin Medical University, Harbin, China; 3 Key Laboratory of Ministry of Education for Gastrointestinal Cancer, Department of Bioinformatics, Fujian Medical University, Fuzhou, China

## Abstract

MicroRNAs (miRNAs) have key roles in breast cancer progression, and their expression levels are heterogeneous across individual breast cancer patients. Traditional methods aim to identify differentially expressed miRNAs in populations rather than in individuals and are affected by the expression intensities of miRNAs in different experimental batches or platforms. Thus it is urgent to conduct miRNA differential expression analysis at an individual level for further personalized medicine research. We proposed a straightforward method to determine the differential expression of each miRNA in an individual patient by utilizing the reversal expression order of miRNA pairs between two conditions (cancer and normal tissue). We applied our method to breast cancer miRNA expression profiles from The Cancer Genome Atlas and two other independent data sets. In total, 292 miRNAs were differentially expressed in individual breast cancer patients. Using the differential expression profile of miRNAs in individual patients, we found that the deregulations of miRNA tend to occur in specific breast cancer subtypes. We investigated the coordination effect between the miRNA and its target, based on the hypothesis that one gene function can be changed by copy number alterations of the corresponding gene or deregulation of the miRNA. We revealed that patients exhibiting an upregulation of hsa-miR-92b and patients with deletions of *PTEN* did not tend to overlap, and hsa-miR-92b and *PTEN* coordinately regulated the pathway of ‘cell cycle' and so on. Moreover, we discovered a new prognostic signature, hsa-miR-29c, whose downregulation was associated with poor survival of breast cancer patients.

## Introduction

MicroRNAs (miRNAs) are short (approximately 22 nt), endogenous non-coding RNAs that regulate gene expression by promoting mRNA degradation or repressing mRNA translation. MiRNAs that function as oncogenes or tumor suppressors are differentially expressed in cancer patients compared with normal samples.^1^ The fold change, *T*-test and significance analysis of microarrays are the most used methods to detect differentially expressed miRNAs in cancer. The fold change is simple but has the obvious disadvantage that it does not provide an estimation of significance. The *T*-test and significance analysis of microarrays rely on the assumption that expression values are normally distributed. These intensity-based methods are also affected by experimental batch effects and data normalization processes.^2^ Some studies have proposed new methods, such as the rank product,^3^ which use the relative order of gene expression values within each sample, considering that the relative order is more robust against batch effects and insensitive to data normalization.^4, 5^ However, these new methods are not appropriate for determining differentially expressed miRNAs in individual breast cancer samples. Differentially expressed miRNAs are highly heterogeneous among individuals of the same cancer type.^6^ Gaire *et al.*^7^ discretized miRNAs based on their expression level rankings in each sample and roughly discretized the first 5% of the miRNAs as low expression and the last 5% as high expression, which lacks statistical estimation.

To avoid these problems, our previous work proposed a method called *RankComp* to detect differentially expressed genes (DEGs) in individual cancer samples using the disrupted ordering of gene expression values in individual cancer sample, based on the observation that the relative ordering of gene expression is overall stable in a particular type of normal tissue but widely disturbed in cancer.^8^ *RankComp* performed well in analyzing mRNA expression profiles, which contain tens of thousands of genes. However, because miRNA expression profiles contain only hundreds of miRNAs, *RankComp* was sensitive to the rank changes of miRNAs in individual patients and not suitable to be applied to individual-level analysis of miRNAs.

The same biological process can be disturbed by the deregulation of miRNA expression or an aberration of their corresponding miRNA targets.^7, 9^ For example, the cell cycle process may become oncogenic by attenuating the tumor-suppressor gene *PTEN* or by elevating the expression of hsa-miR-29b, which can attenuate *PTEN* activity in cancer. *PTEN* and hsa-miR-29b regulate the cell cycle process in a mutually exclusive manner, which indicates that deregulated expression of the miRNA and genomic alterations of its targets do not tend to co-occur in the same patient.^7^ Hence, it is important to detect the deregulation of hsa-miR-29b in individual patients along without alterations of *PTEN*. In our previous study, we discovered that both the deregulation of miRNAs and alterations of *BRCA1/2* could coordinately disrupt the DNA repair process and further affect the overall survival of ovarian cancer patients receiving platinum-based treatment.^9^ Thus it is necessary to develop a method to determine differentially expressed miRNAs in individual patients. Moreover, deregulated miRNAs are considered as diagnostic and prognostic biomarkers because of their significant roles in proliferation, apoptosis and invasion in breast cancer.^10, 11^ Some studies use the average or median score or the expression level as cutoffs to distinguish between high- and low-risk patients.^9, 12, 13, 14^ However, these methods are arbitrary in setting a threshold for prognostic marker detection and are difficult to include in practical application.^15, 16, 17^ To overcome the limitation of these threshold-based methods, it is essential to develop a new method to detect prognostic biomarkers that can be used to obtain prognostic predictions for individual breast cancer patients.^18^

Breast cancer is a complex disease that is characterized by heterogeneity of genetic and epigenetic alterations. Aiming to detect miRNAs with aberrant expression in individual breast cancer patients, we developed a simple and intuitive procedure to determine whether miRNAs are differentially expressed in an individual patient. Given that the expression of miR-B was approximately constant across the cancer and normal samples, the reversal relationship of miR-A and miR-B may be because of the differential expression of miR-A, which can be used as evidence to determine whether miR-A is differentially expressed in individual cancer compared with normal samples. In our analysis, applications of our method were to detect deregulation of miRNAs in specific breast cancer subtypes and miRNA–target pairs with mutually exclusive alterations in breast cancer. Furthermore, based on the individual differential miRNA profiles in breast cancer, we identified that hsa-miR-29c was a new robust prognostic maker for breast cancer, which did not rely on presetting thresholds for prognostic prediction.

## Results

### Identification of reversal miRNA pairs in The Cancer Genome Atlas (TCGA) miRNA training data set

**Figure 1:**
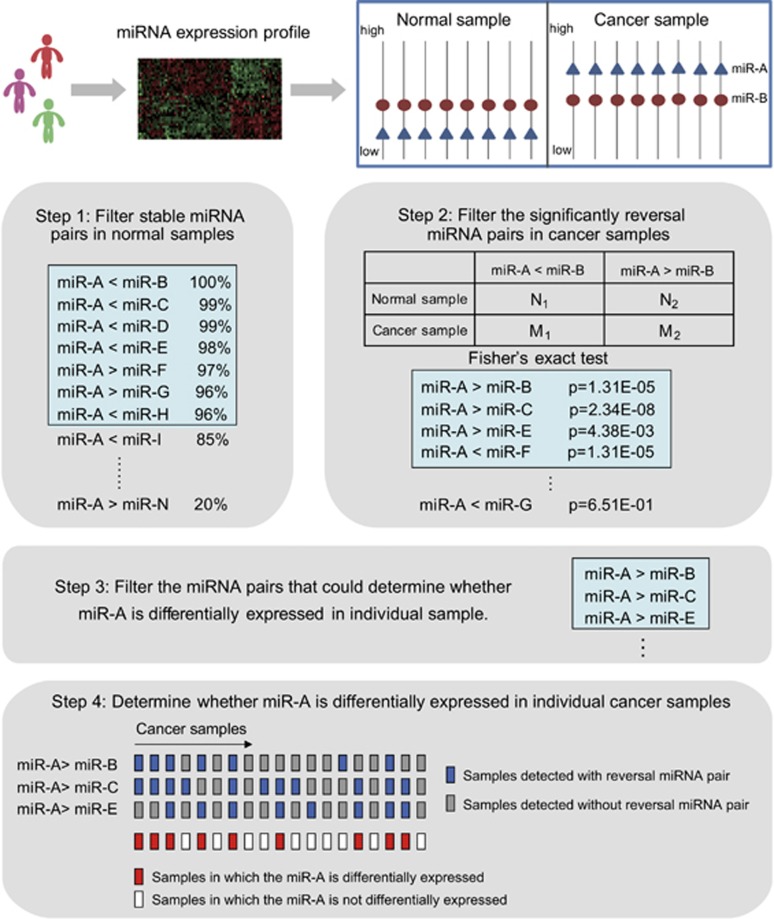
The schematic overview of the analysis procedure.

We distinguished miRNA pairs with stable rank relationships from all pair-wise miRNAs and detected reversal miRNA pairs using Fisher's exact tests across 81 pair-wise breast cancer and normal samples (Table 1). In total, 6872 of the 64 565 stable miRNA pairs were significantly reversed in cancer samples compared with normal samples. According to the filtering criteria (see Materials and methods and Figure 1), we used 676 miRNA pairs to determine the differential expression of 292 miRNAs in individual breast cancer samples in the training data set. For example, the expression value of miR-379 was greater than miR-152, miR-361 and miR-574 in 98.8, 100 and 100% of normal samples, respectively. In cancer samples, however, the expression value of miR-379 was smaller than those of miR-152, miR-361 and miR-574 in 13.6, 35.8 and 7.4% of the samples, respectively, which was significantly different compared with the proportions in normal samples (false discovery rate (FDR)<0.1).

### Performance evaluation in TCGA miRNA testing data set

Using the miRNA reversal pairs derived from the training data set, we determined whether the 292 miRNAs were differentially expressed in individual patients of a testing data set containing 17 pair-wise breast cancer and normal samples (Table 1). Averagely, 53 miRNAs were differentially expressed in each patient, and the average precision was 90.94% for individual breast cancer patients. Each miRNA was detected with differential expression in 3.1 patients on average, and the average precision was 90.97%. In the main text, all results were based on the top three reversal pairs. The top five and seven reversal pairs showed similar results (Table 2). However, when using the *RankComp* method,^8^ only 17.5 miRNAs were differentially expressed in each breast cancer patient on average, and the average precision for each patient was 68.18%. Each miRNA was detected with differential expression in 1.01 breast cancer patients on average, and the average precision for each miRNA was 51.88%. Thus our method performed better at identifying differential expression miRNAs in individual breast cancer samples.

The paired 81 normal samples in the training data set were used to simulate for disease samples. In the training data set, averagely 50 miRNAs, among which 40 miRNAs were upregulated and 10 miRNAs were downregulated, were detected with differential expression in an individual patient by our method. Averagely, 1 miRNA was differentially expressed in 14 patients. Thus 40 and 10 randomly selected miRNAs in each disease sample were separately set to be differentially upregulated and downregulated by adding or subtracting the maximum absolute value of the corresponding miRNA in the original expression values. Each miRNA was set to be differentially expressed in 14 disease samples during the simulation process. The results showed that average values of sensitivity, specificity and F-score were 93.28, 93.36 and 0.9172, respectively. When increasing the number of samples in which each miRNA was differentially expressed, slight changes in sensitivity, specificity and F-score were observed for each scenario (Table 3).

### Breast cancer subtype-specific miRNAs

**Figure 2:**
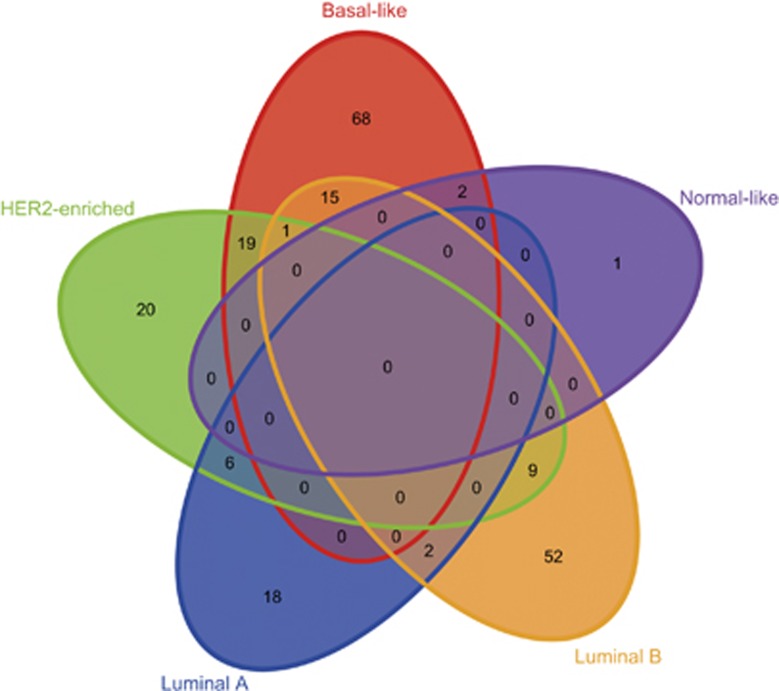
The Venn diagram of subtype-specific miRNAs.

Breast cancer contains many subtypes, including luminal A, luminal B, HER2-enriched, basal-like and normal-like, and the alterations of miRNAs maybe subtype specific.^19, 20^ According to the differential expression profile of miRNAs in individual breast cancer patients and the known subtype labels of breast cancer, we tested whether deregulation of miRNAs tended to be in specific subtypes by hypergeometric distribution model. Totally, we found that 26 miRNAs, 79 miRNAs, 55 miRNAs, 105 miRNAs and 3 miRNAs were significantly altered in luminal A, luminal B, HER2-enriched, basal-like and normal-like, respectively (*P*<0.05, Supplementary Table S1). Some breast cancer subtype-specific miRNAs discovered by us have been confirmed by previous studies. For example, the hsa-miR-106b tended to be upregulated in basal-like breast cancer samples (*P*=1.68 × 10^−8^, hypergeometric test), which has been confirmed by Farazi *et al.*^21^ The hsa-miR-17 was reported as a basal-like subtype-specific miRNA,^22^ and our results showed that the breast cancer patients with upregulation of hsa-miR-17 were significantly enriched in patients with basal-like subtype (*P*=2.22 × 10^−16^). The Venn diagram was used to show the number of miRNAs shared between or among subtypes (Figure 2).

### Coordinated deregulation of miRNAs and copy number alterations of their targets

**Figure 3:**
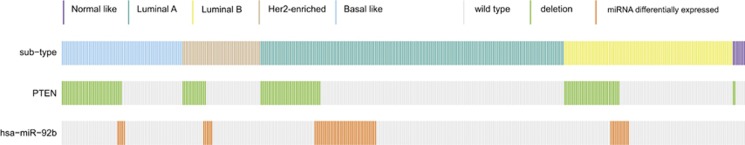
Alterations of hsa-miR-92b and *PTEN* in different subtypes of breast cancer.

Based on the hypothesis that the mutual exclusivity of deregulation expression of miRNA and copy number alteration of its targets could coordinately disrupt the similar pathways, we identified 42 mutually exclusive miRNA–target pairs (Supplementary Table S2), which showed the consistent differential expression of target genes in miRNA deregulated samples and target altered samples when compared with normal samples and significant pathway overlap at *P*<0.05. Here we took the tumor-suppressor gene *PTEN* with deletions in breast cancers as an example. In all, 214 and 154 breast cancer patients carried the deletions of *PTEN* and hsa-miR-92b, respectively. Thirty-five samples were overlapped between the two sample sets, which were significantly less than expected by random chance (*P*=0.022, hypergeometric test, Figure 3). The *PTEN* was significantly downregulated in both the breast cancer samples with upregulation of hsa-miR-92b and the breast cancer samples with deletion of *PTEN* when compared with the normal cohort (*P*=9.53 × 10^−7^ and *P*=2.93 × 10^−16^, *T*-test). The pathway enrichment results revealed that hsa-miR-92b-related DEGs were significantly enriched in 13 pathways (*P*<0.05, hypergeometric test) and that *PTEN*-related DEGs were significantly enriched in 22 pathways (*P*<0.05, hypergeometric test). Four pathways (‘Cell cycle', ‘p53 signaling pathway', ‘Spliceosome' and ‘Oocyte meiosis') were overlapped between the two pathway lists, which cannot be expected by random chance (*P*<0.05, hypergeometric test). Thus the upregulation of hsa-miR-92b and deletions of *PTEN* could coordinately regulate the cell cycle pathway and hence to contribute to the progression of breast cancer. Moreover, we found that deregulation of hsa-miR-92b was luminal A subtype specific (Supplementary Table S1). Thus the mutually exclusive alterations between miRNAs and targets also reflect the mutual exclusivity of breast cancer subtype alterations (Figure 3).

### Prognosis-related differential expression of miRNAs

For each miRNA, 743 TCGA breast cancer patients were divided into two groups: patients with and without differential expression of that miRNA. Cox regression analysis and log-rank tests were used to recognize prognosis-related miRNAs. Our results identified that six miRNAs (hsa-miR-98, hsa-miR-29c, hsa-miR-221, hsa-miR-127, hsa-miR-1224, hsa-miR-99a) were significantly associated with the overall survival of breast cancer patients in the TCGA data set (Supplementary Table S3).

**Figure 4:**
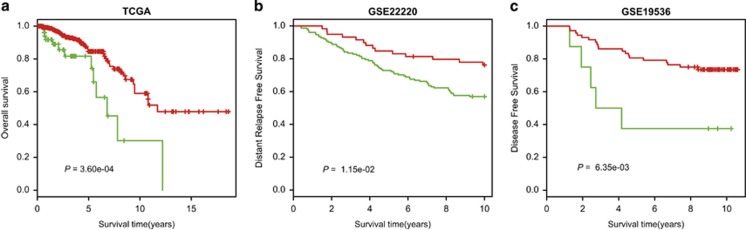
Kaplan–Meier survival curves between patients with and without differential expression of hsa-miR-29c in three data sets. The green and red lines represent patients with and without differential expression of the miRNA, respectively. (**a**) Kaplan-Meier survival curves in the data set of TCGA; (**b**) Kaplan-Meier survival curves in the data set of GSE22220; (**c**) Kaplan-Meier survival curves in the data set of GSE19536.

Among the six miRNAs that have potential prognostic value for breast cancer, only the hsa-miR-29c could be detected differential expression information in two independent data sets (GSE22220 and GSE19536). Thus we took hsa-miR-29c as an example for further analysis. Hsa-miR-29c was differentially downregulated in 148 of the 207 samples in the GSE22220 data set, 9 of the 99 samples in the GSE19536 data set and 55 of the 444 samples in the TCGA data set. For each data set, we further divided the samples into two groups: hsa-miR-29c downregulated group and others. Patients in the ‘hsa-miR-29c differentially downregulated' group displayed significantly shorter median survival time than those in the ‘others' group in the TCGA (*P*=3.60 × 10^−4^, log-rank test, Figure 4), GSE22220 (*P*=1.15 × 10^−2^, log-rank test, Figure 4) and GSE19536 data sets (*P*=6.35 × 10^−3^, log-rank test, Figure 4). According to the PAM50 classification method,^23^ we divided the breast cancer samples into basal-like, HER2-enriched, Luminal A, Luminal B and normal-like subtypes for GSE22220 and GSE19536 data sets, respectively. We tested whether hsa-miR-29c was an independent prognostic marker for breast cancer using multivariate cox regression analysis. When considering other clinical factors, including subtype information, the results of multivariate cox regression analysis showed that hsa-miR-29c exhibited significant association with patient survival in TCGA (hazard ratio (HR)=4.31, 95% confidence interval (CI)=(1.94, 9.60), *P*=3.49 × 10^−4^, Table 4) and the GSE22220 (HR=1.91, 95% CI=(1.04, 3.50), *P*=0.038, Table 4) data set, and was marginally significant in GSE19536 (HR=4.02, 95% CI=(0.77, 21.02), *P*=0.099, Table 4). We also performed the multivariate cox regression analysis on all the other five miRNAs and all of them were significant in TCGA data set (*P*<0.05, Supplementary Table S4). Furthermore, we also used *T*-test to detect DEGs between the hsa-miR-29c downregulated group and others in each data set and performed pathway enrichment analyses in the three data sets. Scrutinizing the top four enriched pathways from the three pathway lists, ‘Cell cycle' and ‘DNA replication' were overlapped, which was not expected by random chance (*P*<0.05, hypergeometric test, Supplementary Table S5).

## Discussion

In this article, we proposed a new rank-based but powerful individual-level method to detect differentially expressed miRNAs. Gene dysfunction can be ascribed to genomic alterations (such as copy number alteration) of the corresponding gene or modification of the expression of the miRNA that attenuates the target gene.^7^ With this approach, it would much easier to combine genomic alterations and differential expressed miRNA at individual level for integrated analysis. Thus one application of our method is to detect mutually exclusive miRNA–target pairs that may coordinately participate in the same pathways. Deregulation of miRNA expression and genomic alterations of its targets may affect the same pathways and result in the same functional regulation, as revealed by the pathway enrichment results in our study. Another application of our method is to identify breast cancer subtype-specific deregulated miRNAs. The results showed that our method could detect some new subtype-specific miRNAs as well as the well-known breast cancer subtype-specific miRNAs, which indicated the reliability of our method.

Moreover, our method can be used to identify prognostic biomarkers for breast cancer. In total, the deregulated expression levels of six miRNAs were associated with overall survival of breast cancers, and hsa-miR-127 has previous evidence for their prognostic effects in breast cancer patients.^24^ Furthermore, hsa-miR-29c was validated as a new robust prognostic biomarker for breast cancer in our study. Patients with hsa-miR-29c downregulation displayed significantly shorter survival than patients without hsa-miR-29c downregulation. Multivariate Cox survival analyses demonstrated that hsa-miR-29c was independent of other clinical factors, including the subtypes, except in the GSE19536 data set, which may be because the majority of samples in this data set (75 of the 99 samples) were censored. Compared with the artificial cutoffs used to identify prognostic biomarkers, our method does not need to accumulate many patients to determine the optimal threshold in practice. In clinical translational application, we only need to compare the expression values of hsa-miR-29c and its reversal miRNA (hsa-miR-30b), which are achieved by our method. Furthermore, we validated that the individual-level differential expression profile of hsa-miR-29c was reliable in the three data sets by the pathway enrichment results. The reproducible pathway enrichment results indicated that downregulation of hsa-miR-29c participated in the pathways of cell cycle and DNA replication by regulating its targets and further affecting breast cancer survival, which deserves further experiments to investigate the detailed mechanism.

Harrell's concordance index (*C*-index) is a popular measure to quantify the predictive accuracy of the prognostic marker and could be used in clinical practice.^25^ A higher *C*-index value than 0.5 indicates a better overall concordance between the predicted risk classification and the observed survival.^26^ Thus we calculated the *C*-index to quantify the predictive accuracy of the miRNAs. The *C*-index values of hsa-miR-29c were 0.602, 0.566 and 0.584 for TCGA, GSE22220 and GSE19536, respectively. *C*-index values of the six miRNAs are presented in Supplementary Table S3. Notably, to identify prognostic signature with higher *C*-index for breast cancer patients, we need to integrate multiple miRNAs that have potential prognostic value for breast cancers and corresponding targets to construct effective classifier, which warrants our future detailed work.

Both our method and the *RankComp* are based on the notion of stable/reversal gene or miRNA pairs to perform individual-level analysis of differentially expressed mRNAs or miRNAs in cancer. Our method gets reversal miRNA pairs from the population of cancer samples. However, the *RankComp* tests whether the stable gene pair is reversal in each individual, which is sensitive to rank changes in small number of gene pairs if the gene list is small, such as the miRNA expression profile. Comparing the results derived from the two methods, our method is suitable to analyze the differential expression of the small amount of miRNAs or genes in individuals, and the *RankComp* is effective in analyzing the expression profiles with large number of genes.

Nevertheless, our present method also has some limitations. First of all, although the positive predictive value, sensitivity, specificity and F-score are relative high, our method may have insufficient power to detect all samples with differential expression of one miRNA. However, for each miRNA, though a certain number of samples with differential expression of the miRNA may be missed, many miRNA–target pairs with mutually exclusive alterations can be identified, which indicates that the identified differentially expressed miRNAs in individual patients captured by our method are true. Moreover, the prognostic marker hsa-miR-29c identified by our method can be reproducible in other two independent data sets. Second, our method depends on sufficient numbers of normal samples to discern the stable relative order of miRNA pairs. To apply our method to other cancer types, one possible way to address this limitation is to collect previously accumulated normal samples from different data sets. Third, the step of calculating variable coefficients for the reversal miRNAs in our method may be weakly affected by the batch effect. However, all the reversal miRNA pairs are obtained based on relative order of expression values, which is robust against batch effects and data normalization. Although all the reversal miRNA pairs could be used to determine the differential expression for the miRNA, to increase the precision, we used the top three miRNAs with smallest variable coefficients. The smaller the variable coefficients of the miRNAs are, the less the results are affected by the batch effect and normalization process. Finally, the miRNAs that are simultaneously detected across different microarray platforms are limited, which seriously affect the number of miRNAs that can be performed individual level analysis for breast cancer. Fortunately, more and more high-throughput next-generation sequencing data are emerging, which can overcome this shortcoming.

## Materials and methods

### Data and preprocessing

Level 3 miRNA expression profiles detected by IlluminaHiSeq and IlluminaGA platforms were obtained from the TCGA data portal (http://tcga-data.nci.nih.gov/tcga/). There were 98 pair-wise breast cancer and normal samples, among which the 81 pair-wise samples from the IlluminaHiSeq platform was set as the training data set and the 17 pair-wise samples from the IlluminaGA platform was used as the testing data set (Table 1). The data set of 743 breast cancer samples without matched normal controls was used for application analysis. The level 3 mRNA expression profiles, including 525 breast cancer samples and 22 normal samples, were detected by Agilent mRNA expression microarrays in TCGA (Table 1). Two independent data sets, GSE19536 and GSE22220 (Table 1), were downloaded from Gene Expression Omnibus. Gene expression levels were quantile normalized and log2 transformed using the R program. The copy number alteration data of breast cancer were downloaded from http://gdac.broadinstitute.org/runs/stddata__2014_01_15/data/BRCA/.^27^ As Mermel *et al.*^28^ did, we used the cutoffs of log2 ratio >0.1 for detecting amplifications and log2 ratio <−0.1 for detecting deletions and got the copy number alteration profile for each gene. Only the genes that showed consistency in copy number alterations and mRNA expression levels (amplification corresponding to higher expression in mRNA and deletion corresponding to lower expression in mRNA compared with the samples without copy number alterations, which were tested by *T*-test with *P*<0.05) were retained for following analysis.

### KEGG pathways

Two hundred and thirty-four pathways were downloaded from the Kyoto Encyclopedia of Genes and Genomes (KEGG, Release 58.0).^29^ These pathways covered 5981 unique genes for pathway enrichment analysis.

### miRNA–target interaction data

In this study, the miRNA–target interaction data set was obtained from the following nine databases: TargetScan,^30^ miRanda,^31^ PITA,^32^ PicTar,^33^ miRBase,^34^ DIANA-microT,^35^ miRTarBase,^36^ miRecords,^37^ and RNAhybrid.^38^ Only miRNA–target interactions appearing in at least two databases were retained.^9^

### Definition of reversal miRNA pairs

The expression values of miRNAs within each sample were ranked in descending order (Figure 1). Each miRNA expression value was converted to its rank within each sample (the smallest expression value corresponding to the minimum rank and the greatest expression value corresponding to the maximum rank). Pair-wise comparisons were performed for all miRNAs to identify miRNA pairs with stable ordering in normal samples. Stable miRNA pairs were defined as patterns of rank in which miR-A<miR-B appeared in >95% of normal samples. Reversal miRNA pairs were defined as miRNA pairs that displayed a significant reversal ordering in cancer samples compared with their stable ordering in normal samples (miR-A<miR-B→miR-A>miR-B) using Fisher's exact test at a FDR<0.1.

### Method work-flow

Step 1: Identification of stable miRNA pairs in normal samples.

Step 2: Identification of reversal miRNA pairs in cancer samples.

Step 3: Two criteria were used to filter the reversal miRNA pairs that could determine whether a miRNA was differentially expressed in an individual patient. We used miR-A as an example. First, only the miRNAs that had the same deregulation directions as miR-A in the miR-A reversal pairs were retained. Here the deregulation directions indicated upregulation or downregulation in the cancer group compared with the normal group. Second, we calculated the variable coefficients in all samples for the miRNAs in the miR-A reversal pairs and ranked these miRNAs by the variable coefficients in increasing order. If there were <3 reversal pairs for miR-A, all were retained; otherwise, only the top 3 reversal pairs were retained for determining whether miR-A was differentially expressed in individual samples. In the main text, the results of the top three miRNA pairs are presented, and similar results of top five and seven miRNA pairs are listed in Table 2. We hypothesized that, if the expression level of miR-B was approximately constant across the cancer and normal samples, the reversal relationship of miR-A and miR-B should occur because of the differential expression of miR-A, which could be used as evidence to determine whether miR-A was differentially expressed in individual cancer samples compared with normal samples.

Step 4: The selected reversal miRNA pairs (miR-A>miR-B, miR-A>miR-C and miR-A>miR-E) were used to determine whether miR-A was differentially expressed in an individual patient in the testing data set. If the pattern of miR-A>miR-B occurred in an individual patient, the rectangle was marked with blue color, otherwise it was marked with gray color. If more than half of reversal miRNA pairs were detected in a patient, we concluded that miR-A was differentially expressed in this individual, which was marked with red color (Figure 1). Notably, the deregulation direction of miR-A was determined from the cancer group compared with the normal group.

### Evaluation of performance

We used the breast cancer samples with matched normal samples to evaluate the performance of our method. If the expression of miR-A in cancer samples was greater than that of matched normal samples, the gold standard deregulation direction of miR-A was upregulated (and vice versa). True positive (TP) represented miRNAs whose deregulation directions were the same as the gold standard, whereas false positive (FP) represented miRNAs whose deregulation directions were judged as the opposite of gold standard. Precision was calculated as positive predictive value: TP/(TP+FP).^39^

Moreover, a simulation was performed to evaluate the performance of our method. To keep the intrinsic structure of real miRNA data, the simulations were conducted based on the real miRNA data set. Here the sensitivity for each miRNA was defined as the ratio of correctly identified samples with differential expression of the miRNA to all samples with differential expression of the miRNA. The specificity for each miRNA was defined as the ratio of correctly identified samples without differential expression of the miRNA to all samples without differential expression of the miRNA. The F-score, a harmonic mean of sensitivity and specificity, was calculated as follows:


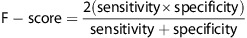


After performing the same simulation 1000 times, the average sensitivity, specificity and F-score values were used to evaluate our method.

### Detection of mutually exclusive miRNA-target pairs with coordinated effects

For each miRNA and its target, a mutually exclusive pair was defined as the differential expression of that miRNA and copy number alterations of its target did not tend to co-occur in the same patient,^7^ which was tested using the hypergeometric distribution (*P*<0.05). Then we used the following criteria to filter the mutually exclusive pairs and find coordinated effects of miRNA expression changes and gene copy number alterations in breast cancer. First, we only retained the mutually exclusive pairs that showed upregulation of miRNA and deletion of its targets or downregulation of miRNA and amplification of its targets. Second, as the miRNA–target pairs have been associated with loss or gain functions of the targets, it should be possible to observe the respective changes in the gene expression as well. Thus we only retained the mutually exclusive pairs that showed consistent expression changes of the targets, which means that the target is significantly downregulated or upregulated in both the samples only with differential expression of the miRNA deregulated samples and the samples only with the copy number alterations of the target when comparing with the normal cohort (*T*-test, *P*<0.05). Finally, for each mutually exclusive miRNA–target pair, based on the hypothesis that differential expression of the miRNA and copy number alterations of its target would affect the same pathway during the progression of breast cancer, we detected DEGs (miRNA-related DEGs) between samples only with differential expression of the miRNA and normal samples and DEGs (target-related DEGs) between samples only with copy number of alterations of the target and normal samples using *T*-test. The genes with FDR<0.05 were defined as DEGs. Then we used the hypergeometric distribution model to test whether the two DEG lists were significantly enriched with pathways derived from the KEGG database. We identified two lists of pathways that were significantly enriched at *P*<0.05. And we used the hypergeometric distribution model to test whether the overlap between the two pathway lists was significant more than expected by chance. Only those mutually exclusively pairs that passed the pathway test were retained.

### Statistical and survival analysis

The survival differences between different groups of patients were estimated using the log-rank test,^40^ and survival curves were plotted using the Kaplan–Meier method.^41^ Cox proportional hazard models were used for univariate and multivariate survival analyses.^42^ Benjamini–Hochberg multiple testing correction was used to estimate the FDR when multiple testing correction was applied.^43^ All statistical analyses were performed using R 3.0.0 (www.bioconductor.org).

## Figures and Tables

**Table 1:** Statistics of the miRNA and mRNA expression data

*Data set*	*Data type*	*Tumor*	*Normal*	*Paired*	*Platform*
TCGA	miRNA	81	81	Y^a^	IlluminaHiSeq_miRNASeq
	miRNA	17	17	Y	IlluminaGA_miRNASeq
	miRNA	743	/	/	IlluminaHiSeq_miRNASeq and IlluminaGA_miRNASeq
	mRNA	525	22	/	Agilent custom 244 K whole-genome microarrays
GSE22220	miRNA	210	/	/	Illumina Human v1 MicroRNA expression beadchip
	mRNA	216	/	/	Illumina humanRef-8 v1.0 expression beadchip
GSE19536	miRNA	101	/	/	Agilent-019118 Human miRNA Microarray 2.0 G4470B
	mRNA	114	/	/	Agilent-014850 Whole Human Genome Microarray 4x44K G4112F

Abbreviations: miRNA, microRNA; TCGA, the cancer genome atlas.

a Y, the data set has the pair-wise tumor and normal samples.

**Table 2:** Summaries of the average precision of samples and miRNAs

*Top pairs*	*TP* *(miRNA)*	*TP+FP* *(miRNA)*	*Precision (miRNA)*	*TP (sample)*	*TP+FP (sample)*	*Precision (sample)*
Top 3 pairs	2.82	3.10	90.97%	48.41	53.23	90.94%
Top 5 pairs	2.52	2.76	91.30%	43.24	47.41	91.20%
Top 7 pairs	2.23	2.44	91.39%	38.35	41.88	91.57%

Abbreviations: FP, false positive; miRNA, microRNA; TP, true positive.

**Table 3:** Sensitivity, specificity and F-score in the simulated data

*Sample number*^a^	*Sensitivity*	*Specificity*	*F-score*
14	0.9328	0.9336	0.9172
20	0.9059	0.9772	0.9328
30	0.8606	0.9787	0.9009
40	0.8521	0.9764	0.8902
50	0.8574	0.9774	0.8955
60	0.8706	0.9768	0.9045

a The number of samples with differential expression of one microRNA.

**Table 4:** Univariate and multivariate Cox regression analyses of the differential expression of hsa-miR-29c and other clinical factors

*Characteristics*	*Univariate analysis*		*Multivariate analysis*	
	*HR (95% CI)*	P*-value*	*HR (95% CI)*	P*-value*
*TCGA*				
hsa-mir-29c	3.68 (1.80, 7.51)	3.56E-04	4.31 (1.94, 9.60)	3.49E-04
ER+ vs ER−	0.67 (0.34, 1.31)	0.24	0.87 (0.25, 3.03)	0.82
PR+ vs PR−	0.58 (0.31, 1.06)	0.076	0.45 (0.17, 1.16)	0.099
Stages 1 and 2 vs ⩾3	2.55 (1.41, 4.62)	1.96E-03	2.53 (1.35, 4.74)	3.68E-03
Age ⩾50 vs <50 years	1.47 (0.76, 2.87)	0.26	1.51 (0.72, 3.17)	0.28
Basal-like vs others	0.92 (0.43, 1.98)	0.83	0.34 (0.08, 1.36)	0.13
Her2-enriched vs others	1.96 (0.87, 4.42)	0.11	0.83 (0.25, 2.76)	0.76
Luminal A vs others	0.58 (0.32, 1.06)	0.077	0.50 (0.22, 1.15)	0.10
Luminal B vs others	1.66 (0.81, 3.43)	0.17	2.97 (0.74, 9.12)	0.13
				
*GSE22220*				
hsa-mir-29c	2.04 (1.14, 3.64)	0.015	1.91 (1.04, 3.50)	0.038
ER+ vs ER−	1.74 (1.03, 2.95)	0.039	2.04 (1.18, 3.53)	0.011
Size <20 vs ⩾20 mm	1.98 (1.14, 3.44)	0.015	1.05 (0.62, 1.77)	0.87
Grades 1 and 2 vs ⩾3	1.52 (0.94, 2.48)	0.090	1.93 (1.11, 3.38)	0.021
Age ⩾50 vs <50 years	0.80 (0.51, 1.26)	0.33	1.29 (0.70, 2.37)	0.42
Basal-like vs others	1.45 (0.84, 2.52)	0.18	2.66 (1.02, 6.95)	0.046
Her2-enriched vs others	1.99 (1.05, 3.76)	0.035	3.14 (1.18, 8.35)	0.022
Luminal A vs others	0.57 (0.35, 0.92)	0.023	1.03 (0.43, 2.48)	0.95
Luminal B vs others	1.87 (1.12, 3.11)	0.017	2.48 (1.01, 6.08)	0.047
				
*GSE19536*				
hsa-mir-29c	4.72 (1.74, 12.79)	2.31E-03	4.02 (0.77, 21.02)	0.099
ER+ vs ER−	0.40 (0.18, 0.90)	0.026	0.48 (0.12, 1.95)	0.30
HER2+ vs HER2−	1.36 (0.50, 3.69)	0.55	3.30 (0.77, 14.18)	0.11
TP53+ vs TP53−	2.99 (1.34, 6.68)	7.74E-03	4.09 (1.23, 13.62)	0.022
Basal-like vs others	1.62 (0.60, 4.33)	0.34	0.17 (0.02, 1.21)	0.076
Her2-enriched vs others	0.85 (0.29, 2.49)	0.77	0.14 (0.02, 1.21)	0.074
Luminal A vs others	0.78 (0.34, 1.79)	0.56	1.15 (0.23, 5.64)	0.86
Luminal B vs others	0.96 (0.29, 3.22)	0.95	0.20 (0.02, 1.74)	0.14

Abbreviations: ER+/−, estrogen receptor positive/negative; HER2+/−, human epidermal growth factor receptor-2 positive/negative; HR, hazard ratio; PR+/−, progesterone receptor positive/negative; TP53+/−, TP53 mutation yes/no.
