# Supplementary material for: Identification of differentially expressed miRNAs in individual breast cancer patient and application in personalized medicine
Source: Oncogenesis. 2016 Feb 15;5(2):e194–. doi: 10.1038/oncsis.2016.4 (PMC5154351; doi:10.1038/oncsis.2016.4)
Supplement: Supplementary Tables [file oncsis20164x1.doc]

**Supplementary Information**

**for**

**Identification of differentially expressed miRNAs in individual breast cancer patient and application in personalized medicine**

**Table of Contents**

**Supplementary Table 1.** Breast cancer sub-type specific miRNAs.

**Supplementary Table 2.** Information of 42 mutually exclusive miRNA-target pairs with coordinated functional effect.

**Supplementary Table 3.** Log-rank test information and C-index values of 6 miRNAs in TCGA dataset.

**Supplementary Table 4.** Univariate and multivariate Cox regression analyses of the differential expression of 5 miRNAs and other clinical factors.

**Supplementary Table 5.** Top 4 enriched pathways ordered by increasing p-value in three datasets.

**Supplementary Table 1. Breast cancer sub-type specific miRNAs.**

| Basal-like | | HER2-enriched | Luminal A | Luminal B | Normal-like |
| --- | --- | --- | --- | --- | --- |
| hsa-let-7b | hsa-miR-500a | hsa-miR-100 | hsa-miR-181d | hsa-let-7c | hsa-mir-147b |
| hsa-let-7d | hsa-miR-500b | hsa-miR-1266 | hsa-miR-182 | hsa-let-7f-1 | hsa-mir-326 |
| hsa-miR-1-2 | hsa-miR-503 | hsa-miR-1269 | hsa-miR-184 | hsa-miR-100 | hsa-mir-339 |
| hsa-miR-100 | hsa-miR-519a-1 | hsa-miR-1270-1 | hsa-miR-190b | hsa-miR-103-2 |  |
| hsa-miR-106a | hsa-miR-539 | hsa-miR-1270-2 | hsa-miR-194-1 | hsa-miR-106a |  |
| hsa-miR-106b | hsa-miR-545 | hsa-miR-1301 | hsa-miR-194-2 | hsa-miR-1180 |  |
| hsa-miR-10a | hsa-miR-556 | hsa-miR-130b | hsa-miR-196a-1 | hsa-miR-1224 |  |
| hsa-miR-1226 | hsa-miR-576 | hsa-miR-142 | hsa-miR-200b | hsa-miR-1247 |  |
| hsa-miR-1229 | hsa-miR-577 | hsa-miR-152 | hsa-miR-222 | hsa-miR-125b-1 |  |
| hsa-miR-1247 | hsa-miR-579 | hsa-miR-183 | hsa-miR-320c-2 | hsa-miR-125b-2 |  |
| hsa-miR-1254 | hsa-miR-582 | hsa-miR-184 | hsa-miR-342 | hsa-miR-1269 |  |
| hsa-miR-1255a | hsa-miR-590 | hsa-miR-187 | hsa-miR-34b | hsa-miR-127 |  |
| hsa-miR-1274b | hsa-miR-636 | hsa-miR-191 | hsa-miR-34c | hsa-miR-128-1 |  |
| hsa-miR-1277 | hsa-miR-652 | hsa-miR-192 | hsa-miR-3607 | hsa-miR-133b |  |
| hsa-miR-1292 | hsa-miR-654 | hsa-miR-194-1 | hsa-miR-378 | hsa-miR-134 |  |
| hsa-miR-1301 | hsa-miR-660 | hsa-miR-194-2 | hsa-miR-381 | hsa-miR-140 |  |
| hsa-miR-1306 | hsa-miR-675 | hsa-miR-195 | hsa-miR-452 | hsa-miR-141 |  |
| hsa-miR-1307 | hsa-miR-758 | hsa-miR-196a-1 | hsa-miR-493 | hsa-miR-145 |  |
| hsa-miR-130b | hsa-miR-766 | hsa-miR-196a-2 | hsa-miR-514-2 | hsa-miR-148b |  |
| hsa-miR-135b | hsa-miR-769 | hsa-miR-210 | hsa-miR-584 | hsa-miR-149 |  |
| hsa-miR-141 | hsa-miR-877 | hsa-miR-2114 | hsa-miR-589 | hsa-miR-151 |  |
| hsa-miR-142 | hsa-miR-885 | hsa-miR-2115 | hsa-miR-598 | hsa-miR-153-1 |  |
| hsa-miR-145 | hsa-miR-9-1 | hsa-miR-2116 | hsa-miR-628 | hsa-miR-154 |  |
| hsa-miR-146a | hsa-miR-9-2 | hsa-miR-218-2 | hsa-miR-653 | hsa-miR-15a |  |
| hsa-miR-150 | hsa-miR-934 | hsa-miR-219-1 | hsa-miR-760 | hsa-miR-15b |  |
| hsa-miR-152 | hsa-miR-942 | hsa-miR-26a-1 | hsa-miR-92b | hsa-miR-16-2 |  |
| hsa-miR-155 |  | hsa-miR-301b |  | hsa-miR-186 |  |
| hsa-miR-17 |  | hsa-miR-3074 |  | hsa-miR-190b |  |
| hsa-miR-181a-2 |  | hsa-miR-30a |  | hsa-miR-191 |  |
| hsa-miR-186 |  | hsa-miR-30c-2 |  | hsa-miR-192 |  |
| hsa-miR-187 |  | hsa-miR-31 |  | hsa-miR-193b |  |
| hsa-miR-188 |  | hsa-miR-3150b |  | hsa-miR-196a-2 |  |
| hsa-miR-18a |  | hsa-miR-335 |  | hsa-miR-197 |  |
| hsa-miR-190 |  | hsa-miR-33a |  | hsa-miR-19a |  |
| hsa-miR-196b |  | hsa-miR-340 |  | hsa-miR-224 |  |
| hsa-miR-19a |  | hsa-miR-345 |  | hsa-miR-2277 |  |
| hsa-miR-19b-1 |  | hsa-miR-3651 |  | hsa-miR-26a-1 |  |
| hsa-miR-19b-2 |  | hsa-miR-3664 |  | hsa-miR-29b-1 |  |
| hsa-miR-200c |  | hsa-miR-3677 |  | hsa-miR-29b-2 |  |
| hsa-miR-20a |  | hsa-miR-381 |  | hsa-miR-301a |  |
| hsa-miR-20b |  | hsa-miR-3917 |  | hsa-miR-30a |  |
| hsa-miR-210 |  | hsa-miR-3934 |  | hsa-miR-3127 |  |
| hsa-miR-2116 |  | hsa-miR-421 |  | hsa-miR-3199-2 |  |
| hsa-miR-219-1 |  | hsa-miR-4326 |  | hsa-miR-32 |  |
| hsa-miR-26a-2 |  | hsa-miR-451 |  | hsa-miR-324 |  |
| hsa-miR-29c |  | hsa-miR-486 |  | hsa-miR-331 |  |
| hsa-miR-301a |  | hsa-miR-487b |  | hsa-miR-338 |  |
| hsa-miR-301b |  | hsa-miR-497 |  | hsa-miR-33b |  |
| hsa-miR-31 |  | hsa-miR-511-2 |  | hsa-miR-342 |  |
| hsa-miR-3170 |  | hsa-miR-551b |  | hsa-miR-34a |  |
| hsa-miR-32 |  | hsa-miR-581 |  | hsa-miR-3613 |  |
| hsa-miR-3200 |  | hsa-miR-589 |  | hsa-miR-3614 |  |
| hsa-miR-320d-1 |  | hsa-miR-590 |  | hsa-miR-3652 |  |
| hsa-miR-326 |  | hsa-miR-659 |  | hsa-miR-3677 |  |
| hsa-miR-330 |  | hsa-miR-766 |  | hsa-miR-369 |  |
| hsa-miR-337 |  |  |  | hsa-miR-375 |  |
| hsa-miR-339 |  |  |  | hsa-miR-379 |  |
| hsa-miR-33a |  |  |  | hsa-miR-3913-1 |  |
| hsa-miR-340 |  |  |  | hsa-miR-3928 |  |
| hsa-miR-345 |  |  |  | hsa-miR-410 |  |
| hsa-miR-3613 |  |  |  | hsa-miR-421 |  |
| hsa-miR-3620 |  |  |  | hsa-miR-423 |  |
| hsa-miR-3651 |  |  |  | hsa-miR-425 |  |
| hsa-miR-3653 |  |  |  | hsa-miR-4326 |  |
| hsa-miR-3664 |  |  |  | hsa-miR-433 |  |
| hsa-miR-3687 |  |  |  | hsa-miR-454 |  |
| hsa-miR-369 |  |  |  | hsa-miR-494 |  |
| hsa-miR-370 |  |  |  | hsa-miR-496 |  |
| hsa-miR-379 |  |  |  | hsa-miR-539 |  |
| hsa-miR-3909 |  |  |  | hsa-miR-550a-2 |  |
| hsa-miR-3917 |  |  |  | hsa-miR-592 |  |
| hsa-miR-3934 |  |  |  | hsa-miR-665 |  |
| hsa-miR-3941 |  |  |  | hsa-miR-676 |  |
| hsa-miR-410 |  |  |  | hsa-miR-7-1 |  |
| hsa-miR-431 |  |  |  | hsa-miR-877 |  |
| hsa-miR-432 |  |  |  | hsa-miR-887 |  |
| hsa-miR-433 |  |  |  | hsa-miR-92a-1 |  |
| hsa-miR-455 |  |  |  | hsa-miR-944 |  |
| hsa-miR-483 |  |  |  | hsa-miR-99a |  |

**Supplementary Table 2.** Information of 42 mutually exclusive miRNA-target pairs with coordinated functional effect.

| miRNA | Target Gene Symbol | Target Geneid | P value |
| --- | --- | --- | --- |
| hsa-mir-200b | PLA2G5 | 5322 | 3.38E-08 |
| hsa-mir-200a | OTUD3 | 23252 | 2.74E-05 |
| hsa-mir-200a | KIF17 | 57576 | 2.74E-05 |
| hsa-mir-200a | EPHA2 | 1969 | 2.74E-05 |
| hsa-mir-760 | KCNC4 | 3749 | 2.84E-04 |
| hsa-mir-760 | CASQ2 | 845 | 2.84E-04 |
| hsa-mir-598 | TCP11L2 | 255394 | 4.00E-03 |
| hsa-mir-429 | PLA2G5 | 5322 | 5.59E-03 |
| hsa-mir-200b | PGM5 | 5239 | 8.83E-03 |
| hsa-mir-200b | TGFBR3 | 7049 | 0.021 |
| hsa-mir-200b | PRPF38B | 55119 | 0.021 |
| hsa-mir-200b | PTBP2 | 58155 | 0.021 |
| hsa-mir-200b | CDC14A | 8556 | 0.021 |
| hsa-mir-200b | EVI5 | 7813 | 0.021 |
| hsa-mir-200b | LPPR4 | 9890 | 0.021 |
| hsa-mir-200b | CD58 | 965 | 0.021 |
| hsa-mir-200b | CNN3 | 1266 | 0.021 |
| hsa-mir-200b | GSTM3 | 2947 | 0.021 |
| hsa-mir-181d | PLA2G5 | 5322 | 0.023 |
| hsa-mir-92b | PTEN | 5728 | 0.024 |
| hsa-mir-34a | TAGLN | 6876 | 0.027 |
| hsa-mir-34a | REXO2 | 25996 | 0.027 |
| hsa-mir-34a | SIDT2 | 51092 | 0.027 |
| hsa-mir-34a | DIXDC1 | 85458 | 0.027 |
| hsa-mir-34a | TRIM29 | 23650 | 0.027 |
| hsa-mir-96 | KLHL32 | 114792 | 0.035 |
| hsa-mir-96 | BACH2 | 60468 | 0.035 |
| hsa-mir-96 | PHIP | 55023 | 0.035 |
| hsa-mir-96 | SLC35A1 | 10559 | 0.035 |
| hsa-mir-96 | CCNC | 892 | 0.035 |
| hsa-mir-96 | LCA5 | 167691 | 0.035 |
| hsa-mir-598 | PDGFA | 5154 | 0.036 |
| hsa-mir-183 | KLHL32 | 114792 | 0.042 |
| hsa-mir-183 | BACH2 | 60468 | 0.042 |
| hsa-mir-183 | REV3L | 5980 | 0.042 |
| hsa-mir-183 | SH3BGRL2 | 83699 | 0.042 |
| hsa-mir-183 | SLC35A1 | 10559 | 0.042 |
| hsa-mir-183 | SNX3 | 8724 | 0.042 |
| hsa-mir-183 | CCNC | 892 | 0.042 |
| hsa-mir-183 | SESN1 | 27244 | 0.042 |
| hsa-mir-183 | ANKRD6 | 22881 | 0.042 |
| hsa-mir-183 | C6orf162 | 57150 | 0.042 |

Note: All the miRNA-target pairs are with up-regulation of miRNAs and deletions of corresponding targets.

**Supplementary Table 3.** Log-rank test information and C-index values of 6 miRNAs in TCGA dataset.

| miRNA | MiRNA Direction^a^ | Log-rank p-value | C-index |
| --- | --- | --- | --- |
| hsa-miR-98 | up | 2.42E-04 | 0.622 |
| hsa-miR-29c | down | 3.60E-04 | 0.602 |
| hsa-miR-221 | down | 6.26E-04 | 0.564 |
| hsa-miR-127 | down | 6.61E-04 | 0.616 |
| hsa-miR-1224 | up | 7.72E-04 | 0.585 |
| hsa-miR-99a | down | 1.12E-03 | 0.613 |

^a^MiRNA Direction means that the miRNA is down-regulated or up-regulated in breast cancer patients.

**Supplementary Table 4.** Univariate and multivariate Cox regression analyses of the differential expression of 5 miRNAs and other clinical factors.

| Characteristics | Univariate analysis | | |  | Multivariate analysis | |
| --- | --- | --- | --- | --- | --- | --- |
|  | HR^a^(95% CI) | P-value | |  | HR(95% CI) | P-value |
| **hsa-miR-1224** | 2.57(1.33,4.97) | | 5.17E-03 |  | 2.69(1.17,6.18) | 0.019 |
| ER^b^+ vs ER- | 0.59(0.31,1.11) | | 0.10 |  | 0.60(0.18,1.95) | 0.39 |
| PR^c^+ vs PR- | 0.53(0.29,0.96) | | 0.036 |  | 0.44(0.17,1.11) | 0.081 |
| Stage1,2 vs >=3 | 2.53(1.41,4.51) | | 1.75E-03 |  | 2.07(1.13,3.79) | 0.018 |
| Age≥50 vs <50 years | 1.30(0.69,2.45) | | 0.41 |  | 1.69(0.83,3.42) | 0.15 |
| Basal-like vs others | 0.89(0.42,1.92) | | 0.77 |  | 0.25(0.05,1.26) | 0.093 |
| HER2-enriched vs others | 1.90(0.85,4.27) | | 0.12 |  | 0.44(0.08,2.37) | 0.34 |
| Luminal A vs others | 0.56(0.31,1.01) | | 0.55 |  | 0.45(0.08,2.34) | 0.34 |
| Luminal B vs others | 1.59(0.78,3.26) | | 0.21 |  | 0.60(0.11,3.45) | 0.57 |
|  |  |  |  |  |  |  |
| **hsa-miR-127** | 2.28(1.13,4.61) | | 0.022 |  | 3.63(1.55,8.50) | 2.90E-03 |
| ER^b^+ vs ER- | 0.59(0.31,1.11) | | 0.10 |  | 0.73(0.22,2.39) | 0.60 |
| PR^c^+ vs PR- | 0.53(0.29,0.96) | | 0.036 |  | 0.45(0.17,1.14) | 0.091 |
| Stage1,2 vs >=3 | 2.53(1.41,4.51) | | 1.75E-03 |  | 2.66(1.41,5.02) | 2.49E-03 |
| Age≥50 vs <50 years | 1.30(0.69,2.45) | | 0.41 |  | 1.51(0.73,3.12) | 0.27 |
| Basal-like vs others | 0.89(0.42,1.92) | | 0.77 |  | 0.21(0.04,1.08) | 0.061 |
| HER2-enriched vs others | 1.90(0.85,4.27) | | 0.12 |  | 0.47(0.09,2.51) | 0.38 |
| Luminal A vs others | 0.56(0.31,1.01) | | 0.55 |  | 0.38(0.07,2.01) | 0.25 |
| Luminal B vs others | 1.59(0.78,3.26) | | 0.21 |  | 0.60(0.10,3.50) | 0.57 |
|  |  |  |  |  |  |  |
| **hsa-miR-221** | 3.10(1.44,6.65) | | 3.74E-03 |  | 4.03(1.74,9.34) | 1.14E-03 |
| ER^b^+ vs ER- | 0.59(0.31,1.11) | | 0.10 |  | 0.78(0.22,2.76) | 0.70 |
| PR^c^+ vs PR- | 0.53(0.29,0.96) | | 0.036 |  | 0.52(0.19,1.34) | 0.17 |
| Stage1,2 vs >=3 | 2.53(1.41,4.51) | | 1.75E-03 |  | 2.12(1.16,3.89) | 0.015 |
| Age≥50 vs <50 years | 1.30(0.69,2.45) | | 0.41 |  | 1.73(0.85,3.52) | 0.13 |
| Basal-like vs others | 0.89(0.42,1.92) | | 0.77 |  | 0.58(0.10,3.25) | 0.53 |
| HER2-enriched vs others | 1.90(0.85,4.27) | | 0.12 |  | 1.21(0.19,7.39) | 0.83 |
| Luminal A vs others | 0.56(0.31,1.01) | | 0.55 |  | 0.70(0.11,4.38) | 0.71 |
| Luminal B vs others | 1.59(0.78,3.26) | | 0.21 |  | 1.27(0.19,8.55) | 0.81 |
|  |  |  |  |  |  |  |
| **hsa-miR-98** | 4.12(1.89,8.95) | | 3.57E-04 |  | 3.13(1.16,8.45) | 0.024 |
| ER^b^+ vs ER- | 0.59(0.31,1.11) | | 0.10 |  | 0.79(0.23,2.73) | 0.71 |
| PR^c^+ vs PR- | 0.53(0.29,0.96) | | 0.036 |  | 0.51(0.19,1.28) | 0.15 |
| Stage1,2 vs >=3 | 2.53(1.41,4.51) | | 1.75E-03 |  | 1.96(1.06,3.61) | 0.030 |
| Age≥50 vs <50 years | 1.30(0.69,2.45) | | 0.41 |  | 1.35(0.66,2.77) | 0.41 |
| Basal-like vs others | 0.89(0.42,1.92) | | 0.77 |  | 0.18(0.03,1.01) | 0.051 |
| HER2-enriched vs others | 1.90(0.85,4.27) | | 0.12 |  | 0.57(0.11,2.99) | 0.51 |
| Luminal A vs others | 0.56(0.31,1.01) | | 0.55 |  | 0.38(0.07,1.97) | 0.25 |
| Luminal B vs others | 1.59(0.78,3.26) | | 0.21 |  | 0.73(0.13,4.11) | 0.72 |
|  |  |  |  |  |  |  |
| **hsa-miR-99a** | 2.97(1.62,5.47) | | 4.64E-04 |  | 4.66(2.09,10.41) | 1.73E-04 |
| ER^b^+ vs ER- | 0.59(0.31,1.11) | | 0.10 |  | 0.55(0.17,1.83) | 0.33 |
| PR^c^+ vs PR- | 0.53(0.29,0.96) | | 0.036 |  | 0.49(0.19,1.24) | 0.13 |
| Stage1,2 vs >=3 | 2.53(1.41,4.51) | | 1.75E-03 |  | 2.65(1.41,4.95) | 2.32E-03 |
| Age≥50 vs <50 years | 1.30(0.69,2.45) | | 0.41 |  | 1.48(0.72,3.01) | 0.28 |
| Basal-like vs others | 0.89(0.42,1.92) | | 0.77 |  | 0.17(0.03,0.86) | 0.034 |
| HER2-enriched vs others | 1.90(0.85,4.27) | | 0.12 |  | 0.39(0.07,2.16) | 0.28 |
| Luminal A vs others | 0.56(0.31,1.01) | | 0.55 |  | 0.36(0.06,1.96) | 0.24 |
| Luminal B vs others | 1.59(0.78,3.26) | | 0.21 |  | 0.34(0.05,2.11) | 0.25 |

^a^HR, hazard ratio; ^b^ER+/-, estrogen receptor positive/negative; ^c^PR+/-, progesterone receptor positive/negative.

Note: Because the number of patients with normal-like sub-type was small, we did not consider the normal-like sub-type during the multivariate cox regression analysis.

**Supplementary Table 5.** Top 4 enriched pathways ordered by increasing p-value in three datasets.

| Dataset | KEGG ID | Pathway name | P value |
| --- | --- | --- | --- |
| TCGA pathway list | hsa04110 | **Cell cycle** | 6.68E-09 |
|  | hsa03030 | **DNA replication** | 3.15E-04 |
|  | hsa04114 | Oocyte meiosis | 1.97E-03 |
|  | hsa03430 | Mismatch repair | 3.22E-03 |
| GSE22220 pathway list | hsa04110 | **Cell cycle** | 1.00E-15 |
|  | hsa04114 | Oocyte meiosis | 3.73E-10 |
|  | hsa04914 | Progesterone-mediated oocyte maturation | 4.11E-05 |
|  | hsa03030 | **DNA replication** | 5.66E-05 |
| GSE19536 pathway list | hsa04110 | **Cell cycle** | 3.66E-13 |
|  | hsa03008 | Ribosome biogenesis in eukaryotes | 1.01E-08 |
|  | hsa03030 | **DNA replication** | 5.96E-08 |
|  | hsa03013 | RNA transport | 1.26E-04 |

Note: The highlighted bold items are overlapped pathways derived from three datasets.
